# Ketamine, an Old–New Drug: Uses and Abuses

**DOI:** 10.3390/ph17010016

**Published:** 2023-12-21

**Authors:** Katarina Savić Vujović, Ana Jotić, Branislava Medić, Dragana Srebro, Aleksandar Vujović, Janko Žujović, Ana Opanković, Sonja Vučković

**Affiliations:** 1 Department of Pharmacology, Clinical Pharmacology and Toxicology, Faculty of Medicine, University of Belgrade, 11129 Belgrade, Serbia; brankicamedic@gmail.com (B.M.); srebrodragana1@gmail.com (D.S.); vuckovicsonja1@gmail.com (S.V.); 2 Clinic for Otorhinolaryngology and Maxillofacial Surgery, University Clinical Center of Serbia, Pasterova 2, 11000 Belgrade, Serbia; anajotic@yahoo.com; 3 Hospital for ENT, KBC “Dragiša Mišović”, 11000 Belgrade, Serbia; aleksandar.vujovic79@gmail.com; 4 Clinical Centre of Montenegro, Centre for Abdominal Surgery, 81000 Podgorica, Montenegro; jankoz@ucg.ac.me; 5 Clinical Centre of Serbia, Clinic for Psychiatry, Faculty of Medicine, University of Belgrade, 11000 Belgrade, Serbia; ana.opankovic@gmail.com

**Keywords:** ketamine, analgesia, anesthesia, clinical implications, abuse

## Abstract

Ketamine as an old–new drug has a variety of clinical implications. In the last 30 years, ketamine has become popular for acute use in humans. Ketamine in standard doses is principally utilized for the induction and maintenance of surgical procedures. Besides its use in anesthesia and analgesia, recent studies have shown that ketamine has found a place in the treatment of asthma, epilepsy, depression, bipolar affective disorders, alcohol and heroin addiction. Ketamine primarily functions as a noncompetitive antagonist targeting the N-methyl-D-aspartate (NMDA) receptor, but its mechanism of action is complex. It is generally regarded as safe, with low doses and short-term use typically not leading to significant adverse effects. Also, ketamine is known as a powerful psychostimulant. During the past decade, ketamine has been one of the commonly abused drugs.

## 1. Introduction

Ketamine (2-chlorohenyl-2-methylamino-cyclohexanone), initially known as “CI-581” in chemical structure, is a phencyclidine (PCP) derivative. Calvin Stevens invented the substance called ketamine in 1962 at the Parke-Davis Pharmaceutical Company [1,2]. Ketamine primarily functions as a noncompetitive antagonist targeting the N-methyl-D-aspartate (NMDA) receptor (NMDAR), but its mechanism of action is complex. Today, ketamine is principally utilized for the induction and maintenance of surgical procedures. Due to its quick induction and rapid recovery, its use has been reported in both veterinary and human surgery. Over the past three decades, ketamine has gained popularity as a preferred choice for parenteral anesthesia in pediatric patients [3]. Ketamine is used in anesthesia in the emergency department but it has shown promising therapeutic potential for the treatment of different disease states, such as depression and asthma. However, due to its properties, dose- and duration-related neurological and peripheral adverse effects are usually reported. Ketamine is known as a powerful psychostimulant, and because of its rewarding and reinforcing effects, it has become a recreational drug, accounting for the steady worldwide increase in its non-medical use [4,5,6].

## 2. Ketamine Pharmacokinetics

Ketamine can be in two isomeric forms, S (+) ketamine and R (−) ketamine. The S (+) isomer is the active enantiomer, and it has several benefits over the R (−) form. It demonstrates a four-fold higher affinity for the NMDA receptor, along with twice the analgesic potency and fewer psychomimetic effects compared to the R (−) isomer. Ketamine is commercially available as a chiral compound consisting of a mixture of both [7]. It is a highly lipid-soluble drug, which accounts for the rapid onset of action as it can easily cross lipid barriers such as the blood–brain barrier. The most efficient administration route is intravenously with a bioavailability of 99%, compared to intramuscular and epidural administration (with bioavailability of 93% and 77%, respectively) [8,9,10]. After intravenous bolus injection, maximum plasma concentration is achieved after 60 s, with a duration of action of 10–15 min. The short duration of a single-dose injection is explained by the rapid redistribution of the drug to other inactive peripheral tissues with high lipid contents (adipose tissue, skeletal muscle, etc.), resulting in a distribution half-life of 7–11 min. The intranasal route of application is easy and promotes fast systemic absorption, due to rich vascularization and the permeability of the nasal mucosa [11,12,13]. Oral administered ketamine has a delayed effect of 15–30 min which is the result of first-pass metabolism in the liver. Norketamine is the main active metabolite which is less potent and is transformed through hydroxylation and conjugation into inactive hydrophilic metabolites that are mainly excreted in urine [14,15].

## 3. Ketamine Pharmacodynamics

Ketamine primarily exerts its analgesic, anesthetic, and psychomimetic effects via antagonism of NMDA receptors in the central nervous system (CNS). In the spinal cord’s dorsal horns, the antagonistic effect on NMDA receptors results in interference with pain transmission, leading to profound analgesia and prevention of central sensitization [16,17]. These effects cumulatively lead to amnestic, analgesic, and dose-dependent anesthetic actions, as well as cataleptic and unique-to-ketamine dissociative states [18]. The primary factor behind the antidepressant effects is the inhibition of NMDA receptors, leading to the activation of α-amino-3-hydroxy-5-methyl-4-isoxazolepropionic acid (AMPA) receptors. AMPA receptor activation induces downstream effects, leading to the upregulation of brain-derived neurotrophic factor (BDNF) and the activation of the signaling receptor tropomyosin receptor kinase B (TrkB) [10].

Furthermore, ketamine triggers the mammalian target of rapamycin (mTOR) pathway, resulting in the deactivation of glycogen synthase kinase 3 (GSK-3) and the inhibition of eukaryotic elongation factor 2 (eEF2) kinase phosphorylation [19,20,21,22]. The active metabolite of ketamine, hydroxynorketamine, does not demonstrate notable interactions with the NMDA receptor. However, it indirectly stimulates AMPA receptors, potentially playing a role in the swift onset of ketamine’s antidepressant effects [23]. According to recent studies, ketamine can have antidepressive effects because it can inhibit the lateral habenula which is known as the “anti-reward center” [24,25]. Ketamine can block muscarinic acetylcholine receptors, voltage-gated calcium channels, and descending monoaminergic pain pathways [26]. The drug exhibits some effects on opioid receptors (mu and kappa) by acting as a partial agonist. Ketamine has the potential to enhance gamma-aminobutyric acid (GABA) synaptic inhibition, stimulate the release of dopamine, and decrease the presynaptic release of glutamate. Certain local anesthetic characteristics are evident, potentially attributable to its capacity to impede neuronal sodium channels. In peripheral areas, ketamine triggers the sympathetic nervous system, manifesting in cardiovascular symptoms such as heightened heart rate, increased cardiac output, and elevated blood pressure by inhibiting catecholamine reuptake. Additionally, ketamine hampers neuronal uptake and elevates serotonergic activity, contributing to nausea and vomiting. Moreover, ketamine prompts catecholamine release and activates β2 adrenergic receptors, resulting in bronchodilation [27,28].

## 4. Clinical Indications

Since ketamine was approved, it has been employed as an anesthetic during diagnostic and surgical procedures, but without muscle relaxation, as an anesthetic for the induction before general anesthetics for its maintenance, and in combination with other anesthetics such as nitrous oxide. It was first used as an anesthetic on American soldiers during the Vietnam War. Over the past 50 years, ketamine has garnered recognition in clinical settings, finding applications in both veterinary and human anesthesia. It continues to be a prominent subject in medical research [29,30,31]. Ketamine has been applied to treat a variety of diseases. It is effective in disorders such as depression, bipolar affective disorder, chronic pain, asthma, and even in the treatment of alcohol and heroin addiction (Table 1).

## 5. Anesthesia

At one point, ketamine was considered suitable for general anesthesia; however, it was quickly revealed to possess a relatively high risk of adverse psychological effects. When recovering from ketamine anesthesia, patients often report unusual symptoms, such as hallucinations, delusions, confusion, “out-of-body” and “near-death” episodes. As a result, ketamine was removed from widespread use in human anesthesia. However, owing to its distinctive properties including potent analgesia, stimulation of the sympathetic nervous system, bronchodilation, and minimal respiratory depression, ketamine and its commercial combinations continue to serve as a significant alternative to other intravenous anesthetics. Consequently, they are extensively utilized in specialized clinics, particularly in pediatrics, psychiatry, and dentistry [61,62,63].

One of the prime candidates for ketamine anesthesia is critical care patients with cardiorespiratory disorders. It should be considered that ketamine, apart from inhibiting the NMDA receptors in postsynaptic neurons in the brain to induce anesthesia, can also increase or maintain cardiac output as well as cerebral blood flow by peripheral inhibition of catecholamine reuptake [32]. It was shown that ketamine in combination with dexmedetomidine induced good efficacy and sedation, less intraoperative use of anesthetics, less arrhythmias and a shorter recovery time in pediatric patients during cardiac catheterizations. A bolus injection which contained ketamine (1 mg/kg) and dexmedetomidine (1 mg/kg) was superior compared to a midazolam–ketamine combination in pediatric patients [64,65]. In adults, for induction, ketamine is given intravenously at doses of 0.5–2 mg/kg, with a maintenance dose of 1–2 mg/min. The doses which are recommended for maintenance vary between 15 and 90 μg/kg/min [33]. Ketamine suppresses respiration to a lesser degree than other anesthetic drugs. Also, it induces bronchodilation and an analgesic effect that is important in patients who have hypersensitive airway diseases. Administering ketamine may pose risks in patients with restricted right ventricular functional reserves and heightened pulmonary vascular resistance [66]. On the contrary, a recent systematic review demonstrated that ketamine use did not affect hemodynamic parameters such as vascular resistance in children with congenital heart disease [67].

Recent studies focused more on using ketamine alone or in combination with local anesthetics in regional anesthesia [68]. It was also shown that ketamine is safer and more effective in regional than in general anesthesia. Its application as an anesthetic agent at the surgical site ensures effective analgesia without notable side effects in children undergoing cleft palate surgery. When employed as a rescue analgesic for promoting a restful sleep pattern and facilitating early resumption of feeding, ketamine surpasses bupivacaine in its effects [34,35]. Additionally, ketamine is commonly integrated as an adjunct drug in pediatric anesthesia for local and regional procedures [34]. Low doses of 0.5 mg/kg ketamine intravenously combined with intravenous diazepam or midazolam, or as a single-drug low-dose ketamine (ranging from 5 to 25 mg/kg/min) can be utilized for sedation and analgesia during local or regional anesthetic procedures [36,69]. Intranasally, ketamine is recommended in pediatric patients whenever it is possible [70].

## 6. Analgesia

Ketamine has been shown to effectively alleviate both acute and chronic pain by inhibiting NMDA receptors in the CNS, thereby preventing the amplification of pain signals. Recent research has reported the immediate analgesic impact of ketamine in pain management. It is used as an analgesic in subanesthetic doses [70]. An intravenous dose of 0.5 or 1 mg/kg applied before surgery provided analgesia after surgery in patients undergoing cholecystectomy and tonsillectomy [37,38,39,40]. Furthermore, suggested regimens involve an initial intravenous bolus of low dosage ranging from 0.25 to 0.5 mg/kg, followed by 50–500 μg/kg/h, for postoperative analgesia and alleviation of exogenous opioid-induced hyperalgesia [41,70]. The use of low ketamine doses is recommended for pain management in the emergency department, administered as a 0.2–0.3 mg/kg bolus over 10 min, followed by an infusion of 0.1–0.3 mg/kg/h, either as an alternative or in addition to commonly used opioids like morphine [42]. Moreover, ketamine stands out as a crucial adjunct during the perioperative period, contributing significantly to achieving desired outcomes when administered according to drug-specific regimens and proven effective dosages. A perioperative combination of ketamine and methadone reduced postoperative pain scores in patients after spinal surgery [71]. A low dose of ketamine applied intravenously with fentanyl reduced pain scores without increasing the side effects in pediatric patients suffering from pectus excavatum [72]. Multimodal approaches such as a combination of ketamine and bupivacaine reduced postoperative pain in patients after total knee arthroplasty [73].

The impact of ketamine on opiate tolerance and hyperalgesia, along with its direct analgesic effects, has promoted a growing trend in its utilization for chronic pain conditions. In a recent investigation, it was demonstrated that intrathecal ketamine infusion promptly alleviates the painful myoclonus in the lower extremities linked to opioid-induced hyperalgesia caused by high-dose intrathecal hydromorphone therapy [74]. Another study presented promising results of the use of ketamine for chronic pain management. Through its effects on the NMDA receptor in the CNS, ketamine inhibited central sensitization and was effective in treating severely ill patients with generalized complex regional pain syndrome (CRPS) [43,75]. Ketamine proved to be highly efficacious as an analgesic for various chronic pain syndromes, encompassing conditions such as chronic pancreatitis pain and post-herpetic neuralgia [76]. Furthermore, it serves as a valuable adjunct to epidural corticosteroid therapy in managing chronic pain, such as chronic lumbar radicular pain [44]. Ketamine is the most effective for pain relief when used in combination with an opioid, and these types of combinations could be particularly useful in managing cancer pain [30,77]. 

## 7. Sedation

Ketamine exerts its sedative effect by enhancing the endogenous antinociceptive system, thereby increasing the descending inhibitory serotonergic pathway [78,79]. The sedative and analgesic effects of ketamine are used in burn patients because of the drug’s ability to maintain respiratory function [80]. Due to the induction of dissociative sedation, the use of a low dose of ketamine in procedural sedation for both adults and children in the emergency department for various painful and emotionally disturbing procedures has gained much support over the last decade. Ketamine’s pharmacokinetic characteristics are not changed due to extracorporeal membrane oxygenation (ECMO) because it is less lipophile than other sedative agents and has minor protein binding [73].

Sedation can be achieved by the intravenous or intramuscular injection of ketamine. In children, ketamine should be used intranasally ranging from 0.5 to 9 mg/kg. Low doses of ketamine combined with propofol provide beneficial analgosedation for short surgical procedures in pediatric emergencies, and in adults undergoing colonoscopy and short gynecological procedures [45,47,48]. In addition, ketamine may be an efficient analgosedation agent in patients in emergency departments. Ketamine has anti-inflammatory effects, stimulates the cardiovascular system and decreases inotropic effects, which makes it convenient for use in septic patients [46]. Evidence demonstrated that in mechanically ventilated patients with intracranial hypertension, ketamine effectively reduced intracranial pressure (ICP) and prevented a rise in ICP during distressing interventions, all without compromising blood or cerebral perfusion pressures. Therefore, the combination of ketamine with benzodiazepine could be used for traumatic brain injury, such as intracranial hypertension in emergencies [81].

Different sedative medications have been studied for safe and effective use in young, uncooperative patients in pediatric dentistry. Ketamine given alone was reported to have side effects and is usually is combined with midazolam to prevent adverse effects and achieve the desired sedative effects [49]. The mixture of ketamine and midazolam, when administered intranasally, produces moderate sedation in pediatric patients who would otherwise require general anesthesia. This combination therapy has also effectively reduced anxiety scores in young patients referred for treatment under general anesthesia [50]. Ketamine in combination with propofol provided better effects in early recovery after gastrointestinal endoscopy [51]. Ketamine in comparison to etomidate was associated with higher survival after urgent endotracheal intubation on the seventh day [52].

## 8. Depression

Depression is the most common chronic and crippling mental illness present in modern-day society associated with high mortality and cost for public health services [53]. It was shown that ketamine rapidly increases the activity of synaptic connections in the cortical and hippocampal neurons and appears to affect consciousness and neuronal changes associated with stress. This is achieved by rapidly inhibiting the NMDA receptor in the CNS by initiating a presynaptic release of glutamate, thereby causing indirect activation of the AMPA receptor in the limbic system. This leads to the activation of mTOR pathways and upregulation of BDNF, which produce changes in synaptic plasticity. This cascade results in enhanced regional activity of the excitation network, including changes in neuroplasticity, which leads to increased synaptogenesis and synaptic potentiation in the limbic system. Another study reported that ketamine’s antidepressive effect through the NMDARs is controversial [82]. The advantage of ketamine compared to other traditional antidepressants is its rapid antidepressive action (for several days) in patients with major depressive disorder (MDD). This is because ketamine bypasses the serotonin pathway and directly leads to increased glutamate levels in the brain [83,84]. Numerous studies examining the impact of ketamine on major depressive disorder indicate a notable and fast alleviation of depressive symptoms following a single ketamine infusion [85,86]. A separate study evaluated the effectiveness of repeated intravenous doses of ketamine in depressed patients, reporting a higher likelihood of relapse in most of the participants [87]. Doses which are used for the treatment of depression in adults are 0.5 or 1 mg/kg [26]. In the pediatric population for the treatment of MDD, effective ketamine antidepressant doses were 0.5 mg/kg intravenously. There was only one study which used doses of 2–7 micrograms/kg [88]. Esketamin was approved by the FDA for the treatment of resistant depression. Also, its intranasal use for patients with major depression and suicidal behavior was approved [89].

## 9. Bipolar Affective Disorder

Bipolar affective disorder has a high rate of suicide as the consequence in about 6% of patients [54]. Therapeutic approaches revolve around the management of symptoms associated with either depression or mania. It was concluded that ketamine improved mood in patients with bipolar depression when it was given sublingually in a period of two to three days or weekly [54]. In another study, ketamine given intravenously showed antidepressive effects in patients with bipolar depression [90]. In children, ketamine given sublingually exhibited significant improvements in mood and behavior [91]. This effect is mainly due to ketamine’s inhibitory action on the NMDA receptor that leads to significant increases in glutamate levels in the cortex [92]. These studies have observed the antidepressant and anti-suicidal effects of ketamine. On the contrary, another study concluded that ketamine did not have an anti-suicidal effect on bipolar disorder and did not demonstrate effectiveness for remission in bipolar depression [93,94].

## 10. Asthma

Asthma is characterized by symptoms of intermittent dyspnea, cough, and wheezing, as a result of bronchial hyperresponsiveness to a variety of stimuli. Typically, therapy is based on inhaled beta-2 agonists, antimuscarinics, and corticosteroids [95]. Ketamine therapy has been researched clinically in the setting of acute asthma attacks. Many studies have shown that ketamine is an effective drug for the treatment status asthmaticus. Mechanical ventilation could be avoided if ketamine was used as adjuvant drug in standard treatment protocols. The first example of its use was observed in a child with a diagnosis of severe asthma. It was reported that intravenous ketamine administration improved symptoms in children with severe asthma. Also, mechanical ventilation was avoided. Gas exchange was improved in mechanically ventilated patients who had remittent bronchospasm after ketamine infusion [96]. These effects could be explained by several mechanisms, which include inhibition of the NMDA receptor in the airways leading to the relaxation of the tracheal smooth muscle and bronchodilation, a decrease in the nitric oxide levels mediating bronchospasm by inhibition of mRNA overexpression, and protein induction by nitric oxide synthetase. As an anti-inflammatory agent, ketamine decreases cytokine production, reduces macrophages, and prevents catecholamine reuptake in the periphery, which increases free norepinephrine levels and produces a more pronounced effect on beta-2 receptors, leading to bronchodilatation [97,98,99,100]. Ketamine can be used in cases of life-threatening status asthmaticus with doses of 0.1–0.2 mg/kg followed by an infusion of 0.15–2.5 mg/kg/h [56,101]. Ketamine as a bronchodilator agent can be used in children with acute asthma exacerbation, but there were not enough studies to give a recommendation about ketamine’s efficacy and time-consuming effects [102]. A recent systematic review showed that there were not enough data on ketamine use in patients with refractory severe asthma exacerbations [55].

## 11. Epilepsy

Glutamate suffusion plays a significant role in epileptic seizures. Consequently, there has been considerable interest in the use of antagonists targeting glutamate receptors, particularly ionotropic NMDA and AMPA receptors, in the treatment of epilepsy [103]. Ketamine has been shown to be promising in treating status epilepticus in patients with prolonged seizures [104]. The combination of drugs such as ketamine and propofol has been given as replacement therapy for seizures due to the clinical effectiveness of electroconvulsive therapy [105,106]. Some data indicated that ketamine protected neurons from glutamate’s harmful effects through NMDA inhibition. A retrospective multicenter cohort study revealed that ketamine is relatively effective and safe for use in treating status epilepticus. In the study (58 patients), permanent control of the treatment of the refractory status epilepticus was achieved in about two-thirds of patients, whereas ketamine’s contribution to permanent control was observed in one-third of episodes [57]. Ketamine can be considered in pediatric patients for the treatment of refractory status epilepticus [58]. Ketamine is a safe, effective, readily available, and cost-effective therapeutic agent for managing refractory status epilepticus in patients with hemodynamic instability.

## 12. Treatment of Alcohol and Heroin Addiction

Addiction is a world health problem with enormous consequences, morbidity and mortality [107,108]. Around 5% of adults globally experience an alcohol use disorder. In the United States, the number of people dying from opioid overdose deaths has increased by 120% between 2010 and 2018 [109,110]. The possible mechanisms of ketamine in treating addiction include increasing neuroplasticity and neurogenesis, obstructing substantial neural structures, curing symptoms of depression, stopping the reconsolidation of memory, inducing mystical experiences, and enhancing the efficiency of the therapy [111]. Ketamine had shown beneficial effects in the treatment of alcohol and heroin addiction [59]. Ketamine in high doses (at a dose of 2.0 mg/kg) in comparison with low doses (with ketamine at 0.2 mg/kg) in heroin addicts incited a full psychedelic experience. Also, high-dose ketamine psychotherapy induces a higher rate of abstinence, reduction in heroin craving, and greater alterations in “nonverbal unconscious” emotional standpoints [111]. In a different randomized study involving heroin-dependent patients, 50% of the group receiving KPT remained abstinent, compared to only 22.2% in the control group. Ketamine was proven to have beneficial effects in proceeding abstinence from alcohol and heroin and was shown to be promising in treating individuals addicted to opioids and cocaine [60].

## 13. Adverse Effects and Interactions of Ketamine

Ketamine is generally considered relatively secure and does not result in serious adverse effects when used at low doses and for short periods. Nevertheless, side effects can occur in up to 40% of patients undergoing continuous subcutaneous infusion of ketamine. These potential effects encompass dizziness, blurred vision, altered hearing, hypertension, nausea and vomiting, vivid dreams, and hallucinations [112,113]. Due to its NMDA receptor-blocking action, ketamine may trigger an excessive release of glutamate and thus cause cortical excitability, which can lead to psychotic behavior and cognitive abnormalities [114]. The immediate effects of ketamine can induce schizophrenia-like symptoms, both positive and negative, in a dose-dependent manner and are mostly related to abnormal activation of the prefrontal cortex and limbic structure. In both fetal and neonatal development, a 5 h exposure may be sufficient to induce a significant neuroapoptotic response because of the brain’s susceptibility to the apoptogenic effects of ketamine. The neuroapoptotic process induced by a ketamine-related NMDA receptor blockade involves bcl-2-like protein 4 (Bax) translocation to mitochondrial membranes and cytochrome c efflux to the mitochondrial outer surface, followed by caspase-3 activation [115].

Repeated doses of ketamine can result in serious toxicity and can induce chronic health problems. Due to ketamine elimination through the urinary tract, symptoms such as increased urinary frequency, urgency, dysuria, hematuria, and cystitis can be present. Ulcerative cystitis which is ketamine-induced can have severe and potentially long-lasting effects on ketamine users. These side effects are seen in clinical practice in individuals who abuse ketamine, and with dose reduction, their incidence can be reduced [116,117,118,119,120]. Animal research has proven that chronic ketamine use can exacerbate neuromuscular strength and nociception. In addition, chronic ketamine administration poses a risk to the mesolimbic, mesocortical, and entorhinostriatal systems. Dysfunction of these neural systems has been connected to several neuropsychiatric disturbances (depression, ADHD, etc.). The neuroapoptotic effects of long-term ketamine use are similar to aging processes and Alzheimer’s disease [121,122,123,124]. Regarding psychiatric side effects, depression was found to worsen over a year in both daily and former ketamine users. Evidence suggests that long-term CNS depression is likely the result of an interaction between ketamine and gabapentin [125,126]. Ketamine as an NMDA receptor blocker reduces neuroplasticity after long-term use. Studies have shown that repeated use of ketamine over a long period of time significantly impairs both short-term and long-term memory, visual recognition, and spatial working memory. However, memory impairment appears to be reversible [127,128,129]. There is minimal evidence establishing a connection between chronic, heavy ketamine use and the diagnosis of a psychotic disorder like schizophrenia. Therefore, the effect of ketamine on psychosis is questionable and needs further research [130,131].

Although ketamine relaxes the bronchial smooth muscle, the drug may result in airway obstruction in 10–20% of pediatric patients, due to the risk of laryngospasm because of heightened salivation [132]. Prolonged ketamine use can result in notable ventricular myocardial apoptosis, fibrosis, and sympathetic denervation, ultimately contributing to the development of cardiac arrhythmia [133]. An increase in liver enzymes has been observed with repeated use of ketamine in chronic pain [134,135]. Additional issues arising from the prolonged administration of ketamine involve intense abdominal pain, dilation of the bile ducts, and bilateral corneal edema [136,137] (Table 2).

Ketamine’s interaction with certain drugs should be noted. Specific agents are described in Table 3. The simultaneous use of ketamine and drugs that reduce liver metabolism by inhibiting CYP3A4 enzymes of the cytochrome P450 enzyme system (i.e., clarithromycin, ketoconazole) can increase the serum levels of ketamine with serious consequences. The administration of ketamine with other CNS depressants such as tramadol, alcohol, and opioids increases the risk of respiratory depression, profound sedation, and coma. Increased neuromuscular blockade is reported after concomitant administration of tubocurarine and atracurium. Lastly, an increased risk of seizure development is seen in patients administered with theophylline. The use of ketamine for general anesthesia or chronic pain in young children and pregnant women should be carefully considered as prolonged exposure can negatively affect fetal or young children’s brain development [138].

## 14. Ketamine Abuse

The first reports of ketamine abuse were described in the late 1960s. During the 1970s and 1980s, extensive abuse was documented in North America, subsequently spreading to Europe and Asia. In the mid-1990s, the use of ketamine as a recreational drug became common in “raves” and nightclubs in Europe and the United States. Over the last decade, Hong Kong and China had widespread abuse of ketamine [111,139,140]. Although ketamine is a controlled substance, its abuse has enlarged in recent years [141]. Ketamine is most commonly used in powder form for inhaling through the nose, but it is also available in liquid and tablet form. Ketamine is mainly known under the names of “special vitamin K”, “super-K”, and “K”. The most appealing aspects of ketamine abuse are “the feeling of melting into the environment”, “visual hallucinations” and “out-of-body experiences” and “laughs” [142]. At elevated doses, ketamine triggers a heightened state of dissociation commonly known as a “K-hole.” In this state, users often undergo intense dissociation, where perceptions become entirely detached from their usual reality [143]. This state of dissociation is unique to the drug and is one of the main reasons for its abuse. Although the full mechanism of ketamine’s dissociative effects is not yet fully understood, there is a link between cognitive impairment and schizophrenia-like symptoms associated with inhibition of the NMDA receptor [144]. Regarding non-medical patterns of ketamine use, a 2009 survey identified 1285 individuals who had used ketamine in the preceding year. When asked about the doses typically used in a session, one-third reported using less than 0.125 g, another third reported using 0.25–0.5 g, and the remaining third reported using more than 1 g in a single session. Additionally, 5% acknowledged regularly using more than 3 g per session. The mean number of consecutive days of ketamine use was 3.5 days, with 11% reporting at least 7 consecutive days [145]. Ketamine is often used with other medications. Another report from an emergency department in Hong Kong showed that patients with acute ketamine toxicity simultaneously used substances such as alcohol, cocaine, and MDMA [146]. Changes in the brain can cause different color perception, disturbances in memory, attention, and cognition. The two most common side effects of ketamine abuse are gastrointestinal and kidney problems. One retrospective study showed that ketamine abusers usually have upper gastrointestinal symptoms, such as an abdominal pain. The cause of the pain may be biliary abnormalities, which can be explained by the smooth muscle relaxation achieved by ketamine’s NMDA receptor blockade. Another common gastrointestinal complication is liver injury [147,148]. Severe symptoms affecting the lower urinary tract (LUTS), such as heightened urination, urinary urgency, and dysuria, which can lead to interstitial cystitis, have been commonly reported in active ketamine users. A connection between long-term ketamine use and the incidence of cystitis is found, but the mechanism is unknown. Also, a link between ketamine abuse and irreversible kidney damage, such as hydronephrosis was reported [149,150,151]. Deaths are rarely associated with ketamine alone, due to its wide therapeutic range [152]. In general, people with acute ketamine toxicity do not need medical attention. Finding rest in a serene environment with minimal auditory and visual stimuli, coupled with sedation, is frequently sufficient, particularly for individuals experiencing hallucinations and other neuropsychological effects. Some agitated and aggressive patients require benzodiazepine therapy such as diazepam or lorazepam. If tachycardia, hallucinations, and hypertension do not improve within 2–3 h with the above treatment, they should be treated accordingly [153,154]. Patients engaging in ketamine abuse do not have a specific treatment. Efforts to address ketamine addiction have focused on modulating the glutamatergic system, given ketamine’s primary pharmacological effects. A recent study indicated that chronic ketamine users receiving lamotrigine, a glutamate release inhibitor, experienced a significant reduction in the frequency and daily dose of ketamine use [1]. Although ketamine is a medical substance with a generally safe pharmacological profile, its abuse carries severe consequences for both individuals and society.

## 15. Conclusions

Ketamine is a non-competitive NMDA receptor blocker that is often used in human and veterinary medicine under strictly controlled conditions as an analgesic and sedative agent. This is a good example of how an existing drug with a relatively stable and established use in clinical practice can be repurposed for multiple uses. The choice of treatment depends on the pathological condition and is effective for diseases such as depression, bipolar disorder, epilepsy, asthma and heroin and alcohol addiction. However, due to its unique dissociative effects, ketamine is often used illegally as a recreational drug, mainly by young adults. Further studies are needed to investigate side effects induced by repetitive use of ketamine, aiming towards developing effective solutions and mitigating these effects.

## Figures and Tables

**Table 1:** Clinical implications and effects of ketamine.

	Effects of Ketamine	
Anesthesia	In adult patients	in adult patients with cardiorespiratory disorders for general anesthesia (induction dose 0.5–2 mg/kg, maintenance dose 1–2 mg/kg/min [32,33]
	In pediatric patients	in pediatric anesthesia for local and regional procedures (doses ranging from 5 to 25 mg/kg/min) [34,35,36]
Analgesia	perioperatively (i.v. 0.5 mg/kg) [37,38,39,40] for postoperative analgesia (i.v. bolus ranging from 0.25 to 0.5 mg/kg, followed by 50–500 μg/kg/h) [41] pain management in emergency departments (i.v. bolus 0.2–0.3 mg/kg over 10 min, followed by i.v. 0.1–0.3 mg/kg/h) [42] for chronic pain (low doses 0.1–0.5 mg/kg) [43,44]	
Sedation	In adult patients	in emergency department and open clinic procedures [45,46]
	In pediatric patients	intranasally in combination with propofol benzodiazepine and midazolam (0.5–9 mg/kg) [45,47,48]
Depression	in major depressive disorder [49,50,51,52,53]	
Bipolar affective disorder	in patients with bipolar depression sublingually [54]	
Asthma	acute asthma attacks [55] statis asthmaticus (0.1–0.2 mg/kg followed by 0.15–2.5 mg/kg/h) [56]	
Epilepsy	in status epilepticus in adult and pediatric patients [57,58]	
Treatment of alcohol and heroin addiction	in heroin and alcohol addicts (high dose of 2.0 mg/kg) [59,60]	

**Table 2:** Adverse effects of ketamine.

	Adverse Effects of Ketamine
Central nervous system	Dizziness [17] Blurred vision [17] Altered hearing [17] Nausea and vomiting [17] Vivid dreams, hallucinations [17] Higher likelihood of depression relapse in patients with repeated intravenous doses [86] Psychotic behavior, schizophrenia-like symptoms [114] Cognitive abnormalities [114] Effects similar to aging processes and Alzheimer’s disease due neuroapoptotic effects of long-term use [120,121,122] Impaired short-term and long-term memory, visual recognition, spatial working memory [125,126,127]
Cardiovascular system	Ventricular myocardial apoptosis and fibrosis [134] Cardiac arrhythmia [134] Arterial hypertension [17]
Respiratory system	Airway obstruction in pediatric patients, due to laryngospasm because of heightened salivation [132,133] Heightened pulmonary vascular resistance [66,67]
Urinary system,	Urinary frequency [116,117,118,119,120] Dysuria [116,117,118,119,120] Hematuria [116,117,118,119,120] Ulcerative cystitis [116,117,118,119,120]
Digestive system	Increase in liver enzymes with repeated use [135,136] Intense abdominal pain [136,137] Dilation of the bile ducts [137]
Eye	Bilateral corneal edema [138]

**Table 3:** Ketamine interaction with certain drugs.

Drugs/Drug Groups	Side Effects/Interactions
Alcohol	Central nervous system depression (respiratory depression, sedation, coma)
Amphetamine/Dextroamphetamine/Lisdexamfetamine	Cardiac effects (arrythmia)
Benzodiazepines (clonazepam, lorazepam)	Central nervous system depression (respiratory depression, sedation, coma)
CYP3A4 inhibitors (ketoconazole, clarithromycin, grapefruit juice)	Increased plasma drug concentrations of ketamine
Doxazosin	Hypotension
Doxepin	Central nervous system depression (respiratory depression, sedation, coma)
Hydroxyzine	Central nervous system depression (respiratory depression, sedation, coma)
Haloperidol	Additive central nervous system effects(dizziness, drowsiness, impairment in thinking)
Mirtazapine	Additive central nervous system effects(dizziness, impairment in thinking, judgment)
Nortriptyline	Central nervous system depression (respiratory depression, sedation, coma)
Neuromuscular blockers (tubocurarine, atracurium)	Neuromuscular blockade
Propranolol	Hypotension
Pregabalin	Central nervous system depression (respiratory depression, sedation, coma)
Trazodone	Central nervous system depression (respiratory depression, sedation, coma)
Theophylline	Increased risk of seizures

## Data Availability

Data sharing is not applicable.

## References

[1] Liu Y., Lin D., Wu B., Zhou W. (2016). Ketamine abuse potential and use disorder. Brain Res. Bull..

[2] Bahji A., Vazquez G.H., Zarate C.A. (2021). Comparative efficacy of racemic ketamine and esketamine for depression: A systematic review and meta-analysis. J. Affect. Disord..

[3] Bokor G., Anderson P.D. (2014). Ketamine: An update on its abuse. J. Pharm. Pract..

[4] Nutt D. (2019). Psychedelic drugs-a new era in psychiatry?. Dialogues Clin. Neurosci..

[5] Aleksandrova L.R., Phillips A.G. (2021). Neuroplasticity as a convergent mechanism of ketamine and classical psychedelics. Trends Pharmacol. Sci..

[6] Sepulveda Ramos C., Thornburg M., Long K., Sharma K., Roth J., Lacatusu D., Whitaker R., Pacciulli D., Moredo Loo S., Manzoor M. (2022). The Therapeutic Effects of Ketamine in Mental Health Disorders: A Narrative Review. Cureus.

[7] Xu J., Lei H. (2014). Ketamine-an update on its clinical uses and abuses. CNS Neurosci. Ther..

[8] Potter D.E., Choudhury M. (2014). Ketamine: Repurposing and redefining a multifaceted drug. Drug Discov. Today.

[9] Hess E.M., Riggs L.M., Michaelides M., Gould T.D. (2022). Mechanisms of ketamine and its metabolites as antidepressants. Biochem. Pharmacol..

[10] Schwenk E.S., Pradhan B., Nalamasu R., Stolle L., Wainer I.W., Cirullo M., Olson A., Pergolizzi J.V., Torjman M.C., Viscusi E.R. (2021). Ketamine in the Past, Present, and Future: Mechanisms, Metabolites, and Toxicity. Curr. Pain Headache Rep..

[11] Moyse D.W., Kaye A.D., Diaz J.H., Qadri M.Y., Lindsay D., Pyati S. (2017). Perioperative Ketamine Administration for Thoracotomy Pain. Pain Physician.

[12] Becker S., Maier A., Peters S., Büttner K., Reiner G. (2021). S-ketamine and intranasal application: Alternatives for the castration of male suckling piglets?. BMC Vet. Res..

[13] Marland S., Ellerton J., Andolfatto G., Strapazzon G., Thomassen O., Brandner B., Weatherall A., Paal P. (2013). Ketamine: Use in anesthesia. CNS Neurosci. Ther..

[14] Kamp J., Jonkman K., van Velzen M., Aarts L., Niesters M., Dahan A., Olofsen E. (2020). Pharmacokinetics of ketamine and its major metabolites norketamine, hydroxynorketamine, and dehydronorketamine: A model-based analysis. Br. J. Anaesth..

[15] Highland J.N., Farmer C.A., Zanos P., Lovett J., Zarate C.A., Moaddel R., Gould T.D. (2022). Sex-dependent metabolism of ketamine and (2R,6R)-hydroxynorketamine in mice and humans. J. Psychopharmacol..

[16] Zanos P., Moaddel R., Morris P.J., Riggs L.M., Highland J.N., Georgiou P., Pereira E.F., Albuquerque E.X., Thomas C.J., Zarate C.A. (2018). Ketamine and Ketamine Metabolite Pharmacology: Insights into Therapeutic Mechanisms. Pharmacol. Rev..

[17] Quibell R., Prommer E.E., Mihalyo M., Twycross R., Wilcock A. (2011). Ketamine. J. Pain Symptom Manag. Ther. Rev..

[18] Kohtala S. (2021). Ketamine–50 years in use: From anesthesia to rapid antidepressant effects and neurobiological mechanisms. Pharmacol. Rep..

[19] Molero P., Ramos-Quiroga J.A., Martin-Santos R., Calvo-Sánchez E., Gutiérrez-Rojas L., Meana J.J. (2018). Antidepressant Efficacy and Tolerability of Ketamine and Esketamine: A Critical Review. CNS Drugs.

[20] Zanos P., Gould T.D. (2018). Mechanisms of ketamine action as an antidepressant. Mol. Psychiatry.

[21] Björkholm C., Monteggia L.M. (2016). BDNF—A key transducer of antidepressant effects. Neuropharmacology.

[22] Castrén E., Kojima M. (2017). Brain-derived neurotrophic factor in mood disorders and antidepressant treatments. Neurobiol. Dis..

[23] Zanos P., Thompson S.M., Duman R.S., Zarate C.A., Gould T.D. (2018). Convergent Mechanisms Underlying Rapid Antidepressant Action. CNS Drugs.

[24] Kim D., Cheong E., Shin H.S. (2018). Overcoming Depression by Inhibition of Neural Burst Firing. Neuron.

[25] Yang Y., Cui Y., Sang K., Dong Y., Ni Z., Ma S., Hu H. (2018). Ketamine blocks bursting in the lateral habenula to rapidly relieve depression. Nature.

[26] Subramanian S., Haroutounian S., Palanca B.J.A., Lenze E.J. (2022). Ketamine as a therapeutic agent for depression and pain: Mechanisms and evidence. J. Neurol. Sci..

[27] Bowdle T.A., Sackett N., Strassman R., Murray T.F., Jelacic S., Chavkin C. (2022). Ketamine Pharmacodynamics Entangled: Comment. Anesthesiology.

[28] Lima T.M., Visacri M.B., Aguiar P.M. (2022). Use of ketamine and esketamine for depression: An overview of systematic reviews with meta-analyses. Eur. J. Clin. Pharmacol..

[29] Wei Y., Chang L., Hashimoto K. (2020). A historical review of antidepressant effects of ketamine and its enantiomers. Pharmacol. Biochem. Behav..

[30] Culp C., Kim H.K., Abdi S. (2021). Ketamine Use for Cancer and Chronic Pain Management. Front. Pharmacol..

[31] Ragnhildstveit A., Slayton M., Jackson L.K., Brendle M., Ahuja S., Holle W., Moore C., Sollars K., Seli P., Robison R. (2022). Ketamine as a Novel Psychopharmacotherapy for Eating Disorders: Evidence and Future Directions. Brain Sci..

[32] Zorumski C.F., Izumi Y., Mennerick S. (2016). Ketamine: NMDA Receptors and Beyond. J. Neurosci..

[33] Peltoniemi M.A., Hagelberg N.M., Olkkola K.T., Saari T.I. (2016). Ketamine: A Review of Clinical Pharmacokinetics and Pharmacodynamics in Anesthesia and Pain Therapy. Clin. Pharmacokinet..

[34] Jha A.K., Bhardwaj N., Yaddanapudi S., Sharma R.K., Mahajan J.K. (2013). A randomized study of surgical site infltration with bupivacaine or ketamine for pain relief in children following cleft palate repair. Paediatr. Anaesth..

[35] Wiegele M., Marhofer P., Lönnqvist P.A. (2019). Caudal epidural blocks in paediatric patients: A review and practical considerations. Br. J. Anaesth..

[36] Stollings J.L., Diedrich D.A., Oyen L.J., Brown D.R. (2014). Rapid-sequence intubation: A review of the process and considerations when choosing medications. Ann. Pharmacother..

[37] Aldamluji N., Burgess A., Pogatzki-Zahn E., Raeder J., Beloeil H. (2021). PROSPECT Working Group collaborators. PROSPECT guideline for tonsillectomy: Systematic review and procedure-specific postoperative pain management recommendations. Anaesthesia.

[38] Bameshki S.A., Salari M.R., Bakhshaee M., Razavi M. (2015). Effect of Ketamine on Post-Tonsillectomy Sedation and Pain Relief. Iran. J. Otorhinolaryngol..

[39] Javid M.J., Hajijafari M., Hajipour A., Makarem J., Khazaeipour Z. (2012). Evaluation of a low dose ketamine in post tonsillectomy pain relief: A randomized trial comparing intravenous and subcutaneous ketamine in pediatrics. Anesth. Pain. Med..

[40] Zhu J., Xie H., Zhang L., Chang L., Chen P. (2018). Efficiency and safety of ketamine for pain relief after laparoscopic cholecystectomy: A meta-analysis from randomized controlled trials. Int. J. Surg..

[41] Meyer-Frießem C.H., Lipke E., Weibel S., Kranke P., Reichl S., Pogatzki-Zahn E.M., Zahn P.K., Schnabel A. (2022). Perioperative ketamine for postoperative pain management in patients with preoperative opioid intake: A systematic review and meta-analysis. J. Clin. Anesth..

[42] Kurdi M.S., Theerth K.A., Deva R.S. (2014). Ketamine: Current applications in anesthesia, pain, and critical care. Anesth. Essays Res..

[43] Taylor S.S., Noor N., Urits I., Paladini A., Sadhu M.S., Gibb C., Carlson T., Myrcik D., Varrassi G., Viswanath O. (2021). Complex Regional Pain Syndrome: A Comprehensive Review. Pain. Ther..

[44] Amr Y.M. (2011). Effect of addition of epidural ketamine to steroid in lumbar radiculitis: One-year follow-up. Pain Physician.

[45] Khutia S.K., Mandal M.C., Das S., Basu S.R. (2012). Intravenous infusion of ketamine-propofol can be an alternative to intravenous infusion of fentanyl-propofol for deep sedation and analgesia in paediatric patients undergoing emergency short surgical procedures. Indian. J. Anaesth..

[46] Ali H., Abdelhamid B.M., Hasanin A.M., Amer A.A., Rady A. (2022). Ketamine-based Versus Fentanyl-based Regimen for Rapid-sequence Endotracheal Intubation in Patients with Septic Shock: A Randomised Controlled Trial. Rom. J. Anaesth. Intensive Care.

[47] Engstrom K., Brown C.S., Mattson A.E., Lyons N., Rech M.A. (2023). Pharmacotherapy optimization for rapid sequence intubation in the emergency department. Am. J. Emerg. Med..

[48] Andolfatto G., Willman E., Joo D., Miller P., Wong W.B., Koehn M., Dobson R., Angus E., Moadebi S. (2013). Intranasal ketamine for analgesia in the emergency department: A prospective observational series. Acad. Emerg. Med..

[49] Oh S., Kingsley K. (2018). Efficacy of Ketamine in Pediatric Sedation Dentistry: A Systematic Review. Compend. Contin. Educ. Dent..

[50] Bahetwar S.K., Pandey R.K., Saksena A.K., Chandra G. (2011). A comparative evaluation of intranasal midazolam, ketamine and their combination for sedation of young uncooperative pediatric dental patients: A triple blind randomized crossover trial. J. Clin. Pediatr. Dent..

[51] Tekeli A.E., Oğuz A.K., Tunçdemir Y.E., Almali N. (2020). Comparison of dexmedetomidine-propofol and ketamine-propofol administration during sedation-guided upper gastrointestinal system endoscopy. Medicine.

[52] Matchett G., Gasanova I., Riccio C.A., Nasir D., Sunna M.C., Bravenec B.J., Azizad O., Farrell B., Minhajuddin A., Stewart J.W. (2022). EvK Clinical Trial Collaborators. Etomidate versus ketamine for emergency endotracheal intubation: A randomized clinical trial. Intensive Care Med..

[53] GBD 2019 Mental Disorders Collaborators (2022). Global, regional, and national burden of 12 mental disorders in 204 countries and territories, 1990–2019: A systematic analysis for the Global Burden of Disease Study 2019. Lancet Psychiatry.

[54] Andrade C. (2017). Ketamine for Depression, 4: In What Dose, at What Rate, by What Route, for How Long, and at What Frequency?. J. Clin. Psychiatry.

[55] Garner O., Ramey J.S., Hanania N.A. (2022). Management of Life-Threatening Asthma: Severe Asthma Series. Chest.

[56] Goyal S., Agrawal A. (2013). Ketamine in status asthmaticus: A review. Indian J. Crit. Care Med..

[57] Gaspard N., Foreman B., Judd L.M., Brenton J.N., Nathen B.R., McCoy B.M., Al-Otaibi A., Kilbride R., Fernández I.S., Mendoza L. (2013). Intravenous ketamine for the treatment of refractory status epilepticus: A retrospective multicenter study. Epilepsia.

[58] Jacobwitz M., Mulvihill C., Kaufman M.C., Gonzalez A.K., Resendiz K., MacDonald J.M., Francoeur C., Helbig I., Topjian A.A., Abend N.S. (2022). Ketamine for Management of Neonatal and Pediatric Refractory Status Epilepticus. Neurology.

[59] Świądro M., Stelmaszczyk P., Lenart I., Wietecha-Posłuszny R. (2021). The Double Face of Ketamine-The Possibility of Its Identification in Blood and Beverages. Molecules.

[60] Edinoff A.N., Wu N.W., Nix C.A., Bonin B., Mouhaffel R., Vining S., Gibson W., Cornett E.M., Murnane K.S., Kaye A.M. (2022). Historical Pathways for Opioid Addiction, Withdrawal with Traditional and Alternative Treatment Options with Ketamine, Cannabinoids, and Noribogaine: A Narrative Review. Health Psychol. Res..

[61] Le Daré B., Pelletier R., Morel I., Gicquel T. (2022). Histoire de la kétamine: Une molécule ancienne qui a toujours la cote [History of Ketamine: An ancientmoleculethatisstillpopulartoday]. Ann. Pharm. Fr..

[62] Barrett W., Buxhoeveden M., Dhillon S. (2020). Ketamine: A versatile tool for anesthesia and analgesia. Curr. Opin. Anaesthesiol..

[63] Nowacka A., Borczyk M. (2019). Ketamine applications beyond anesthesia—A literature review. Eur. J. Pharmacol..

[64] Dhiman T., Verma V., Kumar Verma R., Rana S., Singh J., Badhan I. (2022). Dexmedetomidine-Ketamine or Dexmedetomidine-Midazolam Nebulised Drug Combination as a Premedicant in Children: A Randomised Clinical Trial. Turk. J. Anaesthesiol. Reanim..

[65] Menshawi M.A., Fahim H.M. (2019). Midazolam–ketamine versus dexmedetomidine–ketamine combinations for anesthesia of pediatric patients undergoing cardiac catheterization. Ain-Shams J. Anesthesiol..

[66] Pai A., Heining M. (2007). Ketamine. Contin. Educ. Anaesth. Crit. Care Pain..

[67] Loomba R.S., Gray S.B., Flores S. (2018). Hemodynamic effects of ketamine in children with congenital heart disease and/or pulmonary hypertension. Congenit. Heart Dis..

[68] Prabhakar A., Lambert T., Kaye R.J., Gaignard S.M., Ragusa J., Wheat S., Moll V., Cornett E.M., Urman R.D., Kaye A.D. (2019). Adjuvants in clinical regional anesthesia practice: A comprehensive review. Best. Pr. Res. Clin. Anaesthesiol..

[69] Basturk A., Artan R., Yılmaz A. (2017). Efficacy and safety of midazolam and ketamine in paediatric upper endoscopy. Arab. J. Gastroenterol..

[70] Pansini V., Curatola A., Gatto A., Lazzareschi I., Ruggiero A., Chiaretti A. (2021). Intranasal drugs for analgesia and sedation in children admitted to pediatric emergency department: A narrative review. Ann. Transl. Med..

[71] Murphy G.S., Avram M.J., Greenberg S.B., Benson J., Bilimoria S., Maher C.E., Teister K., Szokol J.W. (2021). Perioperative Methadone and Ketamine for Postoperative Pain Control in Spinal Surgical Patients: A Randomized, Double-blind, Placebo-controlled Trial. Anesthesiology.

[72] Cha M.H., Eom J.H., Lee Y.S., Kim W.Y., Park Y.C., Min S.H., Kim J.H. (2012). Beneficial effects of adding ketamine to intravenous patient-controlled analgesia with fentanyl after the Nuss procedure in pediatric patients. Yonsei Med. J..

[73] Zhang J., Shi K., Jia H. (2018). Ketamine and bupivacaine attenuate post-operative pain following total knee arthroplasty: A randomized clinical trial. Exp. Ther. Med..

[74] Forero M., Chan P.S.L., Restrepo-Garces C.E. (2012). Successful reversal of hyperalgesia/myoclonus complex with low-dose ketamine infusion. Pain Pract..

[75] Duong S., Bravo D., Todd K.J., Finlayson R.J., Tran Q. (2018). Treatment of complex regional pain syndrome: An updated systematic review and narrative synthesis. Can. J. Anaesth..

[76] McMullin P.R., Hynes A.T., Arefin M.A., Saeed M., Gandhavadi S., Arefin N., Eckmann M.S. (2022). Infusion Therapy in the Treatment of Neuropathic Pain. Curr. Pain. Headache Rep..

[77] Aman M.M., Mahmoud A., Deer T., Sayed D., Hagedorn J.M., Brogan S.E., Singh V., Gulati A., Strand N., Weisbein J. (2021). The American Society of Pain and Neuroscience (ASPN) Best Practices and Guidelines for the Interventional Management of Cancer-Associated Pain. J. Pain. Res..

[78] Coles L., Rosenthal E.S., Bleck T.P., Elm J., Zehtabchi S., Chamberlain J., Cloyd J., Shinnar S., Silbergleit R., Kapur J. (2023). Why ketamine. Epilepsy Behav..

[79] Pavlidi P., Megalokonomou A., Sofron A., Kokras N., Dalla C. (2021). Pharmacology of ketamine and esketamine as rapid-acting antidepressants. Psychiatriki.

[80] Gündüz M., Sakalli S., Güneş Y., Kesiktaş E., Ozcengiz D., Işik G. (2011). Comparison of effects of ketamine, ketamine-dexmedetomidine and ketamine-midazolam on dressing changes of burn patients. J. Anaesthesiol. Clin. Pharmacol..

[81] Xu M., Slaughter J.C., Giuse D.A., Patel N., Jordan L.C., Wolf M.S. (2023). Acute Effects of Ketamine on Intracranial Pressure in Children with Severe Traumatic Brain Injury. Crit. Care Med..

[82] Zhou L., Duan J. (2023). The role of NMDARs in the anesthetic and antidepressant effects of ketamine. CNS Neurosci. Ther..

[83] Lapidus K.A., Levitch C.F., Perez A.M., Brallier J.W., Parides M.K., Soleimani L., Feder A., Iosifescu D.V., Charney D.S., Murrough J.W. (2014). A randomized controlled trial of intranasal ketamine in major depressive disorder. Biol. Psychiatry.

[84] Kim J.W., Suzuki K., Kavalali E.T., Monteggia L.M. (2023). Ketamine: Mechanisms and Relevance to Treatment of Depression. Annu. Rev. Med..

[85] Thakurta R.G., Ray P., Kanji D., Das R., Bisui B., Singh O.P. (2012). Rapid antidepressant response with ketamine: Is it thesolution to resistant depression?. Indian. J. Psychol. Med..

[86] Smith-Apeldoorn S.Y., Veraart J.K., Spijker J., Kamphuis J., Schoevers R.A. (2022). Maintenance ketamine treatment for depression: A systematic review of efficacy, safety, and tolerability. Lancet Psychiatry.

[87] Hatchard T., Ortiz A., Owoeye O., Batten L.A., Blier P. (2019). Single, Repeated, and Maintenance Ketamine Infusions for Treatment-Resistant Depression: A Randomized Controlled Trial. Am. J. Psychiatry.

[88] Meshkat S., Rosenblat J.D., Ho R.C., Rhee T.G., Cao B., Ceban F., Danayan K., Chisamore N., Vincenzo J.D.D., McIntyre R.S. (2022). Ketamine use in pediatric depression: A systematic review. Psychiatry Res..

[89] McIntyre R.S., Rosenblat J.D., Nemeroff C.B., Sanacora G., Murrough J.W., Berk M., Brietzke E., Dodd S., Gorwood P., Ho R. (2021). Synthesizing the Evidence for Ketamine and Esketamine in Treatment-Resistant Depression: An International Expert Opinion on the Available Evidence and Implementation. Am. J. Psychiatry.

[90] Permoda-Osip A., Skibisnska M., Bartkowska-Sniatkowska A., Kliwicki S., Chłopocka-Woczniak M., Rybakowski J.K. (2014). Factors connected with efficacy of single ketamine infusion in bipolar depression. Psychiatr. Pol..

[91] Marwaha S., Palmer E., Suppes T., Cons E., Young A.H., Upthegrove R. (2023). Novel and emerging treatments for major depression. Lancet.

[92] Yavi M., Lee H., Henter I.D., Park L.T., Zarate C.A. (2022). Ketamine treatment for depression: A review. Discov. Ment. Health.

[93] Miller J.N., Black D.W. (2020). Bipolar Disorder and Suicide: A Review. Curr. Psychiatry Rep..

[94] Dean R.L., Marquardt T., Hurducas C., Spyridi S., Barnes A., Smith R., Cowen P.J., McShane R., Hawton K., Malhi G.S. (2021). Ketamine and other glutamate receptor modulators for depression in adults with bipolar disorder. Cochrane Database Syst. Rev..

[95] Reddel H.K., Bacharier L.B., Bateman E.D., Brightling C.E., Brusselle G.G., Buhl R., Cruz A.A., Duijts L., Drazen J.M., FitzGerald J.M. (2022). Global Initiative for Asthma Strategy 2021: Executive Summary and Rationale for Key Changes. Am. J. Respir. Crit. Care Med..

[96] Denmark T.K., Crane H.A., Brown L. (2006). Ketamine to avoid mechanical ventilation in severe pediatric asthma. J. Emerg. Med..

[97] Hurth K.P., Jaworski A., Thomas K.B., Kirsch W.B., Rudoni M.A., Wohlfarth K.M. (2020). The Reemergence of Ketamine for Treatment in Critically Ill Adults. Crit. Care Med..

[98] McKinley K., Panakos P., Yousef D. (2021). Characterization of ketamine usage in a large tertiary-care emergency department. Am. J. Emerg. Med..

[99] Xiao S., Zhou Y., Wang Q., Yang D. (2022). Ketamine Attenuates Airway Inflammation via Inducing Inflammatory Cells Apoptosis and Activating Nrf2 Pathway in a Mixed-Granulocytic Murine Asthma Model. Drug Des. Dev. Ther..

[100] Zou H., Wang L.X., Wang M., Cheng C., Li S., Shen Q., Fang L., Liu R. (2019). MTOR-Mediated Autophagy Is Involved in the Protective Effect of Ketamine on Allergic Airway Inflammation. J. Immunol. Res..

[101] La Via L., Sanfilippo F., Cuttone G., Dezio V., Falcone M., Brancati S., Crimi C., Astuto M. (2022). Use of ketamine in patients with refractory severe asthma exacerbations: Systematic review of prospective studies. Eur. J. Clin. Pharmacol..

[102] Binsaeedu A.S., Prabakar D., Ashkar M., Joseph C., Alsabri M. (2023). Evaluating the Safety and Efficacy of Ketamine as a Bronchodilator in Pediatric Patients with Acute Asthma Exacerbation: A Review. Cureus.

[103] Falco-Walter J. (2020). Epilepsy-Definition, Classification, Pathophysiology, and Epidemiology. Semin. Neurol..

[104] Rai S., Drislane F.W. (2018). Treatment of Refractory and Super-refractory Status Epilepticus. Neurotherapeutics.

[105] Erdogan Kayhan G., Yucel A., Colak Y.Z., Ozgul U., Yologlu S., Karlidag R., Ersoy M.O. (2012). Ketofol (mixture of ketamine and propofol) administration in electroconvulsive therapy. Anaesth. Intensive Care.

[106] Tian F., Lewis L.D., Zhou D.W., Balanza G.A., Paulk A.C., Zelmann R., Peled N., Soper D., Santa Cruz Mercado L.A., Peterfreund R.A. (2023). Characterizing brain dynamics during ketamine-induced dissociation and subsequent interactions with propofol using human intracranial neurophysiology. Nat. Commun..

[107] Feltenstein M.W., See R.E., Fuchs R.A. (2021). Neural Substrates and Circuits of Drug Addiction. Cold Spring Harb. Perspect. Med..

[108] Bonnet U., Specka M., Kanti A.K., Scherbaum N. (2022). Differences between users’ and addiction medicine experts’ harm and benefit assessments of licit and illicit psychoactive drugs: Input for psychoeducation and legalization/restriction debates. Front. Psychiatry.

[109] Gowing L.R., Ali R.L., Allsop S., Marsden J., Turf E.E., West R., Witton J. (2015). Global statistics on addictive behaviours: 2014 status report. Addiction.

[110] Skolnick P. (2022). Treatment of overdose in the synthetic opioid era. Pharmacol. Ther..

[111] Ezquerra-Romano I., Lawn W., Krupitsky E., Morgan C.J.A. (2018). Ketamine for the treatment of addiction: Evidence and potential mechanisms. Neuropharmacology.

[112] Vujović K.S., Vučković S., Stojanović R., Divac N., Medić B., Vujović A., Srebro D., Prostran M. (2021). Interactions between Ketamine and Magnesium for the Treatment of Pain: Current State of the Art. CNS Neurol Disord. Drug Targets.

[113] Yu H., Li Q., Wang D., Shi L., Lu G., Sun L., Wang L., Zhu W., Mak Y.T., Wong N. (2012). Mapping the central effects of chronic ketamine administration in an adolescent primate model by functional magnetic resonance imaging (fMRI). Neurotoxicology.

[114] Kruse A.O., Bustillo J.R. (2022). Glutamatergic dysfunction in Schizophrenia. Transl. Psychiatry.

[115] Zhao X., Sun Y., Ding Y., Zhang J., Li K. (2018). miR-34a Inhibitor May Effectively Protect against Sevoflurane-Induced Hippocampal Apoptosis through the Wnt/β-Catenin Pathway by Targeting Wnt1. Yonsei Med. J..

[116] Jhang J.F., Hsu Y.H., Kuo H.C. (2015). Possible pathophysiology of ketamine-related cystitis and associated treatment strategies. Int. J. Urol..

[117] Liu K.M., Chuang S.M., Long C.Y., Lee Y.L., Wang C.C., Lu M.C., Lin R.J., Lu J.H., Jang M.Y., Wu W.J. (2015). Ketamine-induced ulcerative cystitis and bladder apoptosis involve oxidative stress mediated by mitochondria and the endoplasmic reticulum. Am. J. Physiol. Ren. Physiol..

[118] Vu D.M., Freyre K., Opsha O., Opsha Y. (2021). Recreational ketamine-induced cholangiopathy and ulcerative cystitis. Am. J. Emerg. Med..

[119] Niesters M., Martini C., Dahan A. (2014). Ketamine for chronic pain: Risks and benefits. Br. J. Clin. Pharmacol..

[120] Jhang J.F., Hsu Y.H., Jiang Y.H., Kuo H.C. (2016). The Role of Immunoglobulin E in the Pathogenesis of Ketamine Related Cystitis and Ulcerative Interstitial Cystitis: An Immunohistochemical Study. Pain Physician.

[121] Dominguini D., Steckert A.V., Michels M., Spies M.B., Ritter C., Barichello T., Thompson J., Dal-Pizzol F. (2021). The effects of anaesthetics and sedatives on brain inflammation. Neurosci. Biobehav. Rev..

[122] Jelen L.A., Stone J.M. (2021). Ketamine for depression. Int. Rev. Psychiatry.

[123] Garakani A., Murrough J.W., Freire R.C., Thom R.P., Larkin K., Buono F.D., Iosifescu D.V. (2020). Pharmacotherapy of Anxiety Disorders: Current and Emerging Treatment Options. Front. Psychiatry.

[124] Elyassi A.R., Long R.P., Bejnarowicz R.P., Schoneboom B.A. (2011). Possible gabapentin and ketamine interaction causing prolonged central nervous system depression during post-operative recovery following cervical laminoplasty: A case report. J. Med. Case Rep..

[125] Rhee T.G., Shim S.R., Forester B.P., Nierenberg A.A., McIntyre R.S., Papakostas G.I., Krystal J.H., Sanacora G., Wilkinson S.T. (2022). Efficacy and Safety of Ketamine vs Electroconvulsive Therapy Among Patients with Major Depressive Episode: A Systematic Review and Meta-analysis. JAMA Psychiatry.

[126] Radford K.D., Spencer H.F., Zhang M., Berman R.Y., Girasek Q.L., Choi K.H. (2020). Association between intravenous ketamine-induced stress hormone levels and long-term fear memory renewal in Sprague-Dawley rats. Behav. Brain Res..

[127] Price R.B., Duman R. (2020). Neuroplasticity in cognitive and psychological mechanisms of depression: An integrative model. Mol. Psychiatry.

[128] Abbar M., Demattei C., El-Hage W., Llorca P.M., Samalin L., Demaricourt P., Gaillard R., Courtet P., Vaiva G., Gorwood P. (2022). Ketamine for the acute treatment of severe suicidal ideation: Double blind, randomised placebo controlled trial. BMJ.

[129] Morgan C.J., Muetzelfeldt L., Curran H.V. (2010). Consequences of chronic ketamine self-administration upon neurocognitive function and psychological wellbeing: A 1-year longitudinal study. Addiction.

[130] Le T.T., Di Vincenzo J.D., Teopiz K.M., Lee Y., Cha D.S., Lui L.M.W., Rodrigues N.B., Ho R.C., Cao B., Lin K. (2021). Ketamine for psychotic depression: An overview of the glutamatergic system and ketamine’s mechanisms associated with antidepressant and psychotomimetic effects. Psychiatry Res..

[131] Corlett P.R., Honey G.D., Fletcher P.C. (2016). Prediction error, ketamine and psychosis: An updated model. J. Psychopharmacol..

[132] Bayable S.D., Melesse D.Y., Lema G.F., Ahmed S.A. (2021). Perioperative management of patients with asthma during elective surgery: A systematic review. Ann. Med. Surg..

[133] Li Y., Shi J., Yang B.F., Liu L., Han C.L., Li W.M., Dong D.L., Pan Z.W., Liu G.Z., Geng J.Q. (2012). Ketamine-induced ventricular structural, sympathetic and electrophysiological remodelling: Pathological consequences and protective effects of metoprolol. Br. J. Pharmacol..

[134] DeSouza I.S., Thode H.C., Shrestha P., Allen R., Koos J., Singer A.J. (2022). Rapid tranquilization of the agitated patient in the emergency department: A systematic review and network meta-analysis. Am. J. Emerg. Med..

[135] Sear J.W. (2011). Ketamine hepato-toxicity in chronic pain management: Another example of unexpected toxicity or a predicted result from previous clinical and pre-clinical data?. Pain.

[136] Seto W.K., Mak S.K., Chiu K., Vardhanabhuti V., Wong H.F., Leong H.T., Lee P.S.F., Ho Y.C., Lee C.K., Cheung K.S. (2018). Magnetic resonance cholangiogram patterns and clinical profiles of ketamine-related cholangiopathy in drug users. J. Hepatol..

[137] Giroux M.C., Hélie P., Burns P., Vachon P. (2015). Anesthetic and pathological changes following high doses of ketamine and xylazine in Sprague Dawley rats. Exp. Anim..

[138] Wang C., Bhutta A., Zhang X., Liu F., Liu S., Latham L.E., Talpos J.C., Patterson T.A., Slikker W. (2023). Development of a primate model to evaluate the effects of ketamine and surgical stress on the neonatal brain. Exp. Biol. Med..

[139] Yee C.H., Ng C.F., Hong Y.L., Lai P.T., Tam Y.H. (2019). Substance abuse effects on urinary tract: Methamphetamine and ketamine. Hong Kong Med. J..

[140] Le T.T., Cordero I.P., Jawad M.Y., Swainson J., Di Vincenzo J.D., Jaberi S., Phan L., Lui L.M.W., Ho R., Rosenblat J.D. (2022). The abuse liability of ketamine: A scoping review of preclinical and clinical studies. J. Psychiatr. Res..

[141] Palamar J.J., Salomone A., Rutherford C., Keyes K.M. (2021). Extensive Underreported Exposure to Ketamine Among Electronic Dance Music Party Attendees. J. Gen. Intern. Med..

[142] Orhurhu V.J., Vashisht R., Claus L.E., Cohen S.P. (2023). Ketamine Toxicity. StatPearls [Internet].

[143] Craig C.L., Loeffler G.H. (2014). The ketamine analog methoxetamine: A new designer drug to threaten military readiness. Mil. Med..

[144] Zanos P., Moaddel R., Morris P.J., Georgiou P., Fischell J., Elmer G.I., Alkondon M., Yuan P., Pribut H.J., Singh N.S. (2016). NMDAR inhibition-independent antidepressant actions of ketamine metabolites. Nature.

[145] Winstock A.R., Mitcheson L., Gillatt D.A., Cottrell A.M. (2012). The prevalence and natural history of urinary symptoms among recreational ketamine users. Br. J. Urol. Int..

[146] Li C., Lai C.K., Tang M.H.Y., Chan C.C.K., Chong Y.K., Mak T.W.L. (2019). Ketamine analogues multiplying in Hong Kong. Hong Kong Med. J..

[147] Sassano-Higgins S., Baron D., Juarez G., Esmaili N., Gold M. (2016). A review of ketamine abuse and diversion. Depress. Anxiety.

[148] Wong G.L., Tam Y.H., Ng C.F., Chan A.W., Choi P.C., Chu W.C., Lai P.B., Chan H.L., Wong V.W. (2014). Liver injury is commonamong chronic abusers of ketamine. Clin. Gastroenterol. Hepatol..

[149] Cheung R.Y., Lee J.H., Chan S.S., Liu D.W., Choy K.W. (2014). A pilot study of urine cytokines in ketamine-associated lower urinary tract symptoms. Int. Urogynecol. J..

[150] Chen C.L., Wu S.T., Cha T.L., Sun G.H., Meng E. (2022). Molecular Pathophysiology and Potential Therapeutic Strategies of Ketamine-Related Cystitis. Biology.

[151] Abuse C.K., Tran V.H., Nelson M., Nogar J., Bramante R.M. (2014). Bilateral hydronephrosis and cystitis resulting from. West. J. Emerg. Med..

[152] Mihaljević S., Pavlović M., Reiner K., Ćaćić M. (2020). Therapeutic Mechanisms of Ketamine. Psychiatr. Danub..

[153] Chan Y.C. (2021). Correction to: Acute and Chronic Toxicity Pattern in Ketamine Abusers in Hong Kong. J. Med. Toxicol..

[154] Saad M., Le Clec’h B., Dhonneur G. (2020). Hypoalbuminemia-Related Prolonged Sedation After General Anesthesia: A Case Report. A A Pract..

